# Myofilament Calcium Sensitivity: Mechanistic Insight into TnI Ser-23/24 and Ser-150 Phosphorylation Integration

**DOI:** 10.3389/fphys.2016.00567

**Published:** 2016-12-15

**Authors:** Hussam E. Salhi, Nathan C. Hassel, Jalal K. Siddiqui, Elizabeth A. Brundage, Mark T. Ziolo, Paul M. L. Janssen, Jonathan P. Davis, Brandon J. Biesiadecki

**Affiliations:** Department of Physiology and Cell Biology and Davis Heart and Lung Research Institute, Ohio State UniversityColumbus, OH, USA

**Keywords:** cardiac troponin I, phosphorylation, calcium sensitivity, functional integration

## Abstract

Troponin I (TnI) is a major regulator of cardiac muscle contraction and relaxation. During physiological and pathological stress, TnI is differentially phosphorylated at multiple residues through different signaling pathways to match cardiac function to demand. The combination of these TnI phosphorylations can exhibit an expected or unexpected functional integration, whereby the function of two phosphorylations are different than that predicted from the combined function of each individual phosphorylation alone. We have shown that TnI Ser-23/24 and Ser-150 phosphorylation exhibit functional integration and are simultaneously increased in response to cardiac stress. In the current study, we investigated the functional integration of TnI Ser-23/24 and Ser-150 to alter cardiac contraction. We hypothesized that Ser-23/24 and Ser-150 phosphorylation each utilize distinct molecular mechanisms to alter the TnI binding affinity within the thin filament. Mathematical modeling predicts that Ser-23/24 and Ser-150 phosphorylation affect different TnI affinities within the thin filament to distinctly alter the Ca^2+^-binding properties of troponin. Protein binding experiments validate this assertion by demonstrating pseudo-phosphorylated Ser-150 decreases the affinity of isolated TnI for actin, whereas Ser-23/24 pseudo-phosphorylation is not different from unphosphorylated. Thus, our data supports that TnI Ser-23/24 affects TnI-TnC binding, while Ser-150 phosphorylation alters TnI-actin binding. By measuring force development in troponin-exchanged skinned myocytes, we demonstrate that the Ca^2+^ sensitivity of force is directly related to the amount of phosphate present on TnI. Furthermore, we demonstrate that Ser-150 pseudo-phosphorylation blunts Ser-23/24-mediated decreased Ca^2+^-sensitive force development whether on the same or different TnI molecule. Therefore, TnI phosphorylations can integrate across troponins along the myofilament. These data demonstrate that TnI Ser-23/24 and Ser-150 phosphorylation regulates muscle contraction in part by modulating different TnI interactions in the thin filament and it is the combination of these differential mechanisms that provides understanding of their functional integration.

## Introduction

Contraction and relaxation of the heart is fundamentally dependent on the conversion of the dynamic rise and fall in intracellular cytosolic Ca^2+^ into force production (Kobayashi and Solaro, 2005). The binding of Ca^2+^ to troponin (Tn) activates the myofilament allowing the interaction of myosin with actin, the generation of force and contraction. Calcium regulation of the myofilament through this binding event is not a simple “on and off” switch (Solzin et al., 2007; Davis and Tikunova, 2008; Biesiadecki and Davis, 2014; Davis et al., 2016). Rather, activation and deactivation are both active processes that involve a number of dynamic and complex protein-protein interactions (Manning et al., 2011). Each of these dynamic interactions have the potential to modulate similar and/or different myofilament contraction and relaxation functions (Biesiadecki et al., 2014; Chung et al., 2016; Janssen et al., 2016). Thus, the myofilament response to Ca^2+^ can be intricately manipulated through Tn to regulate cardiac function and improve resulting in an improved outcome in cardiac disease (Li et al., 2010; Alves et al., 2014; Shettigar et al., 2016).

The phosphorylation of troponin I (TnI) represents a key mechanism in modulating the myofilament response to Ca^2+^ (Solaro et al., 1976; Solaro and Kobayashi, 2011; Liu et al., 2014; Nixon et al., 2014). One of the most physiologically and pathologically relevant Tn phosphorylations is the adrenergic mediated protein kinase A phosphorylation of TnI at serines 23 and 24 (Ser-23/24). TnI Ser-23/24 phosphorylation contributes significantly to Ca^2+^ regulation of the myofilament, resulting in decreased Ca^2+^-sensitive force production and accelerated myofilament relaxation (Kranias and Solaro, 1982; de Tombe and Stienen, 1995; Herron et al., 2001; Kentish et al., 2001; Sakthivel et al., 2005; Biesiadecki et al., 2007; Ramirez-Correa et al., 2010). In addition to Ser-23/24, TnI can undergo phosphorylation on at least 12 additional residues as the end result of different signaling pathways (Zhang et al., 2012). A large subset of literature has described the effects many of these phosphorylations impart on cardiac function through altering the Ca^2+^ sensitive regulation of force production in isolation. Although a single phosphorylation is sufficient to alter cardiac function, the heart simultaneously contains multiple TnI phosphorylations that can be independently altered in response to physiological and pathological stress (Pi et al., 2003; Nixon et al., 2014; Lang et al., 2015). Therefore, it is not the isolated effect of a single TnI phosphorylation that is responsible for overall cardiac regulation, but rather the simultaneous presence of multiple TnI phosphorylations that combine to exhibit expected or unexpected functional integration. The significance of these multiple phosphorylations results in a functional integration as demonstrated by the finding that the contractile effects of TnI Ser-23/24 phosphorylation are dependent on the myofilament phosphorylation background (Biesiadecki et al., 2007; Kooij et al., 2010; Nixon et al., 2012; Salhi et al., 2014; Lang et al., 2015). The mechanisms underlying how combined TnI phosphorylations integrate to regulate the myofilament response to Ca^2+^ remain poorly understood.

The integrated function of multiple TnI phosphorylations is significant in cardiac disease (Zhang et al., 2012). We have demonstrated that the phosphorylation of TnI at Ser-23/24 and Ser-150 are both significantly increased in response to ischemic conditions following a myocardial infarction (Nixon et al., 2014). At first glance this seems paradoxical in that Ser-23/24 phosphorylation decreases while Ser-150 phosphorylation increases Ca^2+^-sensitivity (Nixon et al., 2012; Oliveira et al., 2012). However, the combination of Ser-23/24 and Ser-150 phosphorylation exhibit functional integration by differentially regulating cardiac function from that of either phosphorylation alone, retaining contractile force (normal Ca^2+^ sensitivity) but accelerating relaxation (accelerated Ca^2+^ dissociation) (Nixon et al., 2012, 2014). This and others' findings demonstrate the significance of TnI phosphorylation integration to cardiac function during disease (Kooij et al., 2010; Boontje et al., 2011; Taglieri et al., 2011; Salhi et al., 2014). Ultimately, elucidating the different molecular mechanisms utilized during simultaneous TnI phosphorylation will provide insight into the regulatory protein interactions that can be therapeutically targeted, such as by altered phosphorylation, to modulate contraction and relaxation in cardiac disease.

In the current study, we sought to investigate the molecular mechanisms utilized by Ser-23/24 and Ser-150 phosphorylation to modulate contraction both individually and when integrated. Based on our previous work, we hypothesized that Ser-23/24 and Ser-150 differentially alter the binding affinities of TnI within the thin filament to elicit functional integration. By employing mathematical modeling and protein-protein binding experiments, we identified that Ser-23/24 or Ser-150 pseudo-phosphorylation each modulate different TnI binding affinities in the thin filament. TnI Ser-150 pseudo-phosphorylation decreases the affinity of TnI for actin, which is unaffected by Ser-23/24 pseudo-phosphorylation. Through force-Ca^2+^ measurements in Tn-exchanged skinned myocytes we demonstrate that the magnitude of pseudo-phosphorylation-dependent Ca^2+^ sensitive force development is directly related to the amount of TnI pseudo-phosphorylation present at Ser-23/24 or Ser-150. We further demonstrate that these pseudo-phosphorylations function similarly regardless of whether they are present on the same or different TnI molecules. These data demonstrate that TnI Ser-23/24 and Ser-150 phosphorylations affect distinct TnI interactions in the thin filament to result in differential regulation of the steady-state and kinetics of contraction.

## Materials and methods

### Mathematical model

The thin filament interactions responsible for the steady-state TnC Ca^2+^ binding and kinetic Ca^2+^ dissociation from TnC for each phosphorylated TnI were determined by solving the mathematical model described by Siddiqui et al. (2016). Briefly, this model describes Ca^2+^ mediated thin filament regulation based on six biochemical states of TnC. The TnC states are described by a series of first order differential equations. Reaction rate constants for each TnI phosphorylation were solved using Scilab computation to approximate the previously determined Tn and thin filament biochemical determinants (Ca^2+^-sensitivity and Ca^2+^ dissociation Nixon et al., 2012, 2014) for each TnI phosphorylation.

### cDNA constructs

All cardiac TnI amino acid residue numbers presented in this manuscript are given according to the human sequence including the first methionine. Site-directed mutagenesis (QuickChange Lightning, Agilent, Santa Clara, CA) was conducted according to manufacturer's instructions to generate cDNA constructs encoding human TnI pseudo-phosphorylations: Ser-150 to Asp (S150D), Ser-23/24 to Asp (S23/24D), Ser-23/24 to Asp with Ser-150 to Asp (S23/24/150D). All resultant constructs were verified by DNA sequencing.

### Protein expression and purification

Plasmids encoding the individual recombinant human cardiac Tn subunits were transformed and expressed in *Escherichia coli* and purified to homogeneity as previously described (Sumandea et al., 2003; Kobayashi et al., 2005; Kobayashi and Solaro, 2006; Nixon et al., 2012). Cardiac Tn complexes were prepared and reconstituted by sequential dialysis as previously described (Kobayashi and Solaro, 2006; Biesiadecki et al., 2007; Nixon et al., 2014).

### Solid-phase protein binding

ELISA-based solid-phase protein binding assays were conducted as previously described to determine the effect of phosphorylation to alter TnI binding to actin compared to that of non-phosphorylated TnI (Biesiadecki and Jin, 2011). Briefly, 2 μM F-actin dissolved in Buffer A (in mM: 150 KCl, 3 MgCl_2_, 10 MOPS, pH 7.0) was used to coat a 96-well microtiter plate in 100 μL/well at 4°C overnight. Unbound actin was washed with Buffer T (Buffer A containing 0.1% Tween-20). Following washes, the wells were blocked with Buffer A containing 1% BSA. Following removal of blocking solution, serial dilutions of TnI WT or phosphomimetics (S23/24D, S150D, or S23/24/150D) were incubated in 100 μL/well for 2 h at room temperature. The wells were then washed and bound TnI was quantified by ELISA using a mouse anti-cardiac TnI antibody (Fitzgerald; clone C5) and appropriate HRP-conjugated secondary antibody. Following the addition of H_2_O_2_-ABTS (2,2′-azino-bis(3-ethylbenzthiazoline-6-sulphonic acid) substrate solution, the absorbance at 405 nm was monitored over the linear course of color development. Protein binding assays were conducted in triplicate wells and repeated. Each assay contained wells that were coated with F-actin but incubated with buffer in the absence of TnI as a negative control.

### Myocyte force production

All animal protocols and procedures were performed in accordance with National Institutes of Health guidelines and approved by the Institutional Laboratory Animal Care and Use Committee at The Ohio State University. Steady-state Ca^2+^-sensitive force development was measured in Tn-exchange permeabilized rat myocytes as described previously (Salhi et al., 2014). Briefly, following mechanical isolation from frozen rat ventricular tissue, cardiac myocyte preparations were skinned by resuspension in relaxing solution (in mM; 97.92 KOH, 6.24 ATP, 10 EGTA, 10 Na_2_CrP, 47.58 Kprop, 6.54 MgCl_2_, 100 BES, pH 7.0) containing 1% peroxide-free Triton X-100 (Anapoe-X-100, Anatrace, Maumee, OH) with incubation at room temperature for 10 min rocking. Following skinning, myocytes were centrifuged and immediately resuspended for Tn exchange.

Exchange of exogenous recombinant human cardiac Tn into skinned rat myocytes was performed as described previously by incubating myocytes in exchange buffer (in mM; 200 KCl, 5 MgCl_2_, 1 DTT, 5 EGTA, 20 MOPS, pH 6.5) containing 13 uM column purified Tn overnight at 4°C (Sumandea et al., 2003; Biesiadecki et al., 2007; Nixon et al., 2012). Exchange with Tn WT, Tn S23/24D, Tn S150D, and Tn S23/24/150D groups was conducted by incubating skinned myocytes overnight in exchange solution containing the single purified Tn complex indicated. Exchange with Tn WT+S23/24D, Tn WT+S150D, and Tn S23/24D+S150D groups was conducted by incubating skinned myocytes overnight in exchange solution containing an equal molar ratio of the two purified indicated Tn complexes to a final total Tn of 13 uM. Calcium regulated force development in skinned Tn exchanged rat ventricular myocyte preparations was performed similar to that previously described (Salhi et al., 2014). Briefly, Tn exchanged myocytes were attached to two micro-needles using aquarium sealant (Marineland, Noblesville, IN) and sarcomere length was adjusted to 2.2 um by visualization on a calibrated monitor. The perfusion pipette of a constant perfusion control system (VC-8M Eight Channel Mini-Valve Perfusion System, Warner Instruments, Hamden, CT) was then placed close to the myocyte such that the outflow perfused the entire myocyte. Experiments were performed by flowing a series of activating mixtures consisting of relaxing and activating solution over the myocyte. Activating solution was identical in composition to relaxing solution but containing varied free Ca^2+^ concentration (pCa 10.0 to 4.5) (Fabiato and Fabiato, 1979). Developed force was measured at each activating perfusion followed by perfusion with relaxing solution. The activating developed force was subtracted from the subsequent relax perfused force measure. Time-dependent force rundown was determined by comparison of the first developed maximal force to the force developed upon final maximal activation at the end of the experiment. Any cell exhibiting greater than 20% force rundown was discarded. Cell cross-sectional area was calculated after the force experiment on a calibrated monitor as previously described (Salhi et al., 2014). Force-pCa curves were fit using a modified Hill-equation (Biesiadecki et al., 2007; Nixon et al., 2012). Experiments were conducted at room temperature. Data were acquired with custom-made LabView software and analyzed using Igor Pro.

### Protein electrophoresis and western blot

Skinned myocytes were solubilized in denaturing buffer (2% SDS, 0.1% bromophenol blue, 10% glycerol and 50 mM Tris-HCl, pH 6.8), heated for 5 min at 80°C and clarified by centrifugation for 5 min. SDS-PAGE and Western blot were carried similar to that previously described (Biesiadecki et al., 2010; Liu et al., 2012; Nixon et al., 2012). Briefly, proteins were separated on 12% (29:1) Laemmli gel and transferred to PVDF. Following blocking with 1% BSA in TBS resultant membranes were incubated with a mouse anti-cardiac TnI antibody (Fitzgerald; clone C5). Following washes, membranes were incubated with a Dylight labeled fluorescent secondary antibody (Jackson ImmunoResearch Laboratories, Inc, West Grove, PA) and visualized on a Typhoon 9410 imager (GE Healthcare, Piscataway, NJ). Differential migration of endogenous mouse TnI versus exogenous exchanged human TnI allowed for quantification by densitometry analysis. In groups where two different Tn complexes were exchanged the amount of total exogenous incorporation was determined and the exchange of the two complexes considered similar.

### Data processing and statistical analysis

Data are presented as mean ± the standard error of the mean. Protein binding and developed force was plotted against TnI concentration or Ca^2+^, respectively, and fit with a logistic sigmoid function mathematically equivalent to the Hill equation to determine 50% maximal binding or force as previously described. Results of Tn exchange and force development experiments were compared by One-way ANOVA with Tukey's *post-hoc* test. *p* < 0.05 was considered statistically significant.

## Results

### The functional integration of TnI Ser-23/24 and Ser-150 phosphorylations is determined by their differential protein interactions within the thin filament

**Figure 1:**
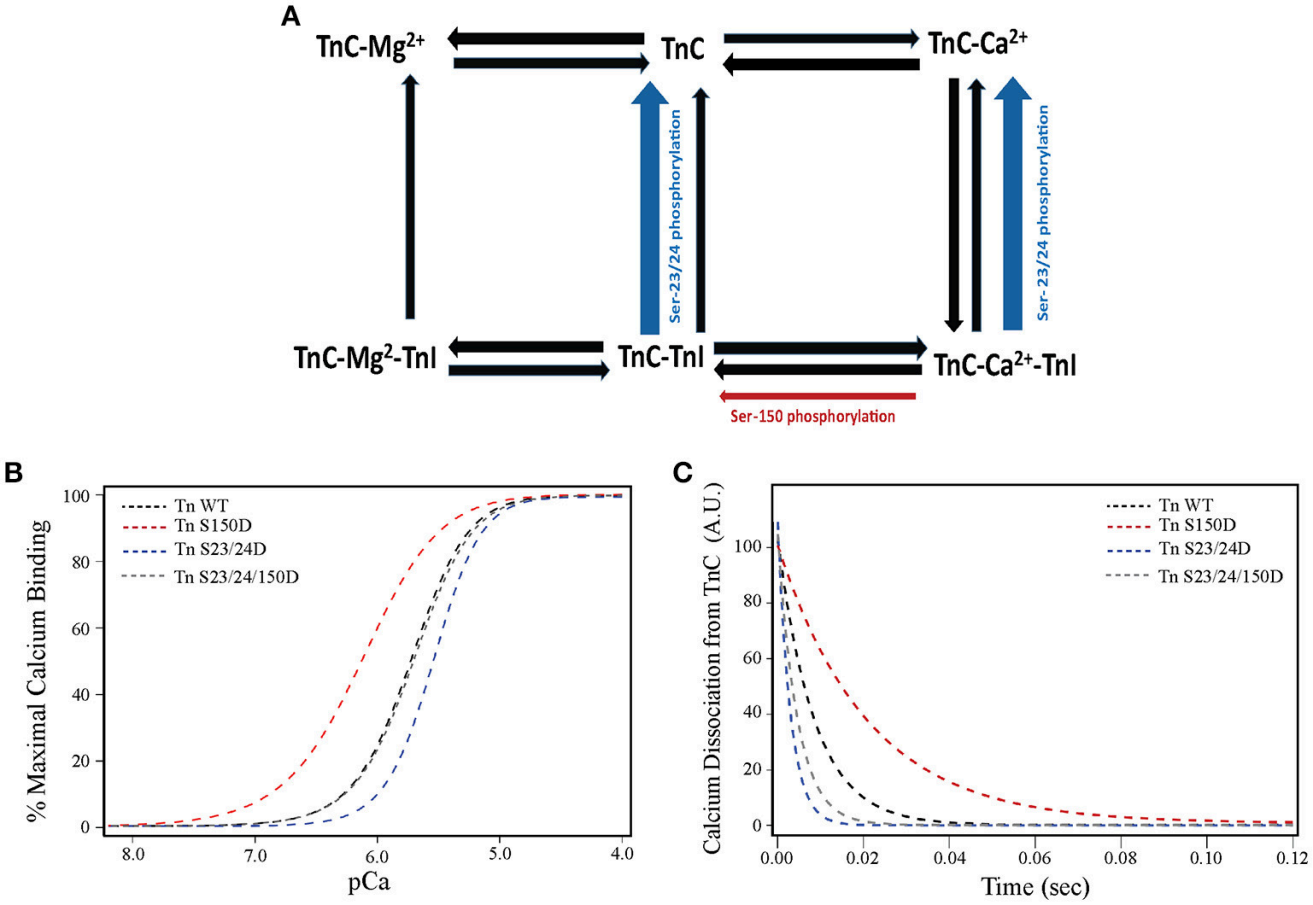
**TnI Ser-23/24 and Ser-150 differentially alter thin filament activation through different TnI mediated mechanisms. (A)** Diagrammatic representation of the biochemical states in the Siddiqui et al. model (Siddiqui et al., 2016). The rates altered to fit our previous biochemical data for thin filaments containing TnI Ser-150 (red arrow) and Ser-23/24 phosphorylation (blue arrow) are identified. Model simulation of **(B)** steady-state Ca^2+^-binding and **(C)** Ca^2+^ dissociation from TnC in human thin filaments containing WT (Tn WT, black dashed line), Ser-150 pseudo-phosphorylated (Tn S150D, red dashed line), Ser-23/24 pseudo-phosphorylated (Tn S23/24D, blue dashed line) or Ser-23/24 and Ser-150 pseudo-phosphorylated (Tn S23/24/150D, gray dashed line) TnI demonstrating a similar effect of these phosphorylations to our previously reported biochemical measurements (Nixon et al., 2014).

Our previous studies demonstrate functional integration of TnI phosphorylations exhibit different contractile function than predicted from the effect of each phosphorylation in isolation (Nixon et al., 2012, 2014; Salhi et al., 2014). Functional integration of Ser-150 with Ser-23/24 phosphorylation restores WT-like Ca^2+^-sensitivity (normal force) while maintaining accelerated thin filament Ca^2+^ dissociation (accelerated relaxation) (Nixon et al., 2012, 2014). We first investigated if altered thin filament protein interactions within a single regulatory unit were sufficient to explain this functional integration. Toward this end we employed a mathematical model based on several biochemical states of TnC (apo or bound to Ca^2+^, Mg^2+^, and/or TnI) recently developed by the Davis laboratory to identify mechanisms involved in TnI phosphorylation integration (Siddiqui et al., 2016). Changes in the hill coefficient and cooperativity are not incorporated into the model. This model identified that Ser-23/24 and Ser-150 phosphorylations differentially modulate regulation of contraction by altering the affinity of different TnI thin filament interactions. By simply accelerating the dissociation of TnI from TnC (Figure 1A), the model recapitulates the biochemical effects of TnI Ser-23/24 phosphorylation on steady-state Ca^2+^ binding and Ca^2+^ dissociation in thin filament and the isolated Tn complex (Figures 1B,C). Our model further asserts that Ser-150 phosphorylation utilizes a separate mechanism by slowing the dissociation of Ca^2+^ from TnC-TnI and increasing the availability of TnI for TnC (Figure 1A). Making only these two alterations are sufficient to accurately model the increase in Ca^2+^ sensitivity and decrease in Ca^2+^ dissociation induced by Ser-150 phosphorylation in both the Tn and thin filament state (Figures 1B,C). Based upon these results we hypothesized that modulation of these separate TnI binding affinities is sufficient to reconcile TnI Ser-23/24 and Ser-150 phosphorylation functional integration. Indeed, the model demonstrates that combination of these two phosphorylation mechanisms is sufficient to recapitulate the Ser-150 induced blunting of Ser-23/24 desensitization while maintaining accelerated thin filament Ca^2+^ dissociation in both isolated Tn and the thin filament (Figures 1B,C), similar to our previously published data for that of Tn S23/24/150D (Nixon et al., 2014, 2012).

**Figure 2:**
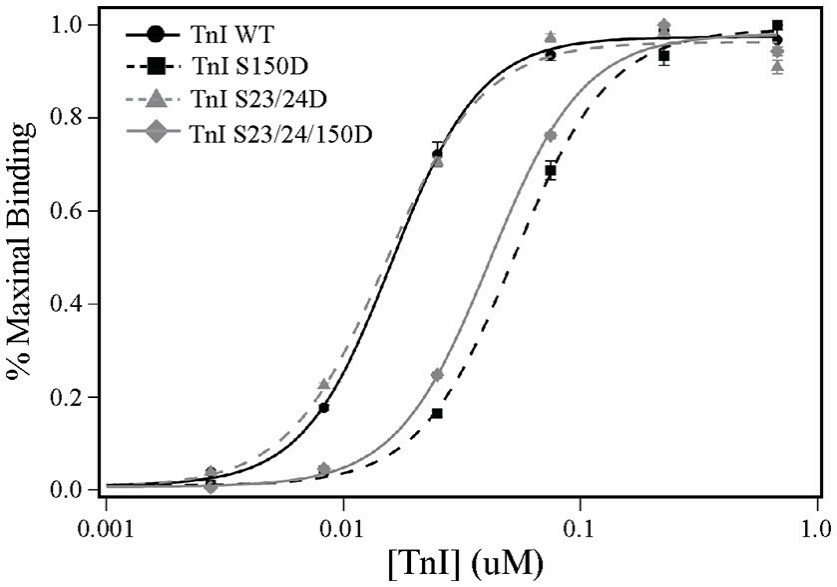
**TnI Ser-150, but not Ser-23/24, pseudo-phosphorylation decreases TnI binding to actin**. Average solid-phase TnI-actin protein binding data demonstrating that compared to WT (TnI WT: black circle, solid line), the pseudo-phosphorylation of Ser-150 (TnI S150D: black Square, dashed line) and Ser-23/24/150 (TnI S23/24/150D: gray diamond, solid line) decrease TnI binding to actin while the pseudo-phosphorylation of Ser-23/24 (TnI S23/24D: gray triangle, dashed line) do not. Protein binding assays were conducted in triplicate wells and repeated.

TnI availability in the model is dependent on several interactions TnI may have in the thin filament and allows us to predict the molecular mechanisms responsible for Tn mediated modification alteration of thin filament regulation. To mechanistically understand the bases of TnI phosphorylation effects predicted by the model, we employed a solid-phase protein binding assay to measure the affinity of cardiac TnI for actin under different pseudo-phosphorylated states. Protein binding experiments demonstrate that while purified cardiac Ser-23/24 pseudo-phosphorylated TnI does not alter the actin binding affinity, Ser-150 pseudo-phosphorylation decreases TnI-actin binding by 3.3-fold (TnI concentration required for 50% maximum binding to actin: TnI WT = 16.0 ± 0.5 nM, TnI S23/24D = 15.0 ± 0.4 nM, TnI S150D = 52.9 ± 2 nM; TnI WT vs. TnI S150D = *p* < 0.05) (Figure 2). According to the model, we hypothesized that the Ser-150-mediated decrease in TnI actin affinity would be maintained upon integration with Ser-23/24 phosphorylation. Our protein binding data confirms that TnI S23/24/150D still exhibits a 2.6-fold decrease in actin binding affinity compared to WT TnI (TnI concentration required for 50% maximum binding to actin: TnI S23/24/150D = 41.2 ± 0.7 nM; TnI WT vs. TnI S23/24/150D = *p* < 0.05) (Figure 2). This decreased affinity of TnI Ser-150 pseudo-phosphorylation, but not Ser-23/24 pseudo-phosphorylation for actin demonstrates that Ser-23/24 and Ser-150 phosphorylation can differentially modulate the binding of TnI to actin, validating model predictions that Ser-23/24/150 functional integration is at least in part based on different molecular mechanisms of the two phosphorylations to modulate TnI interactions within the thin filament.

### Functional integration of TnI Ser-23/24 and Ser-150 pseudo-phosphorylation in rat myocytes

**Figure 3:**
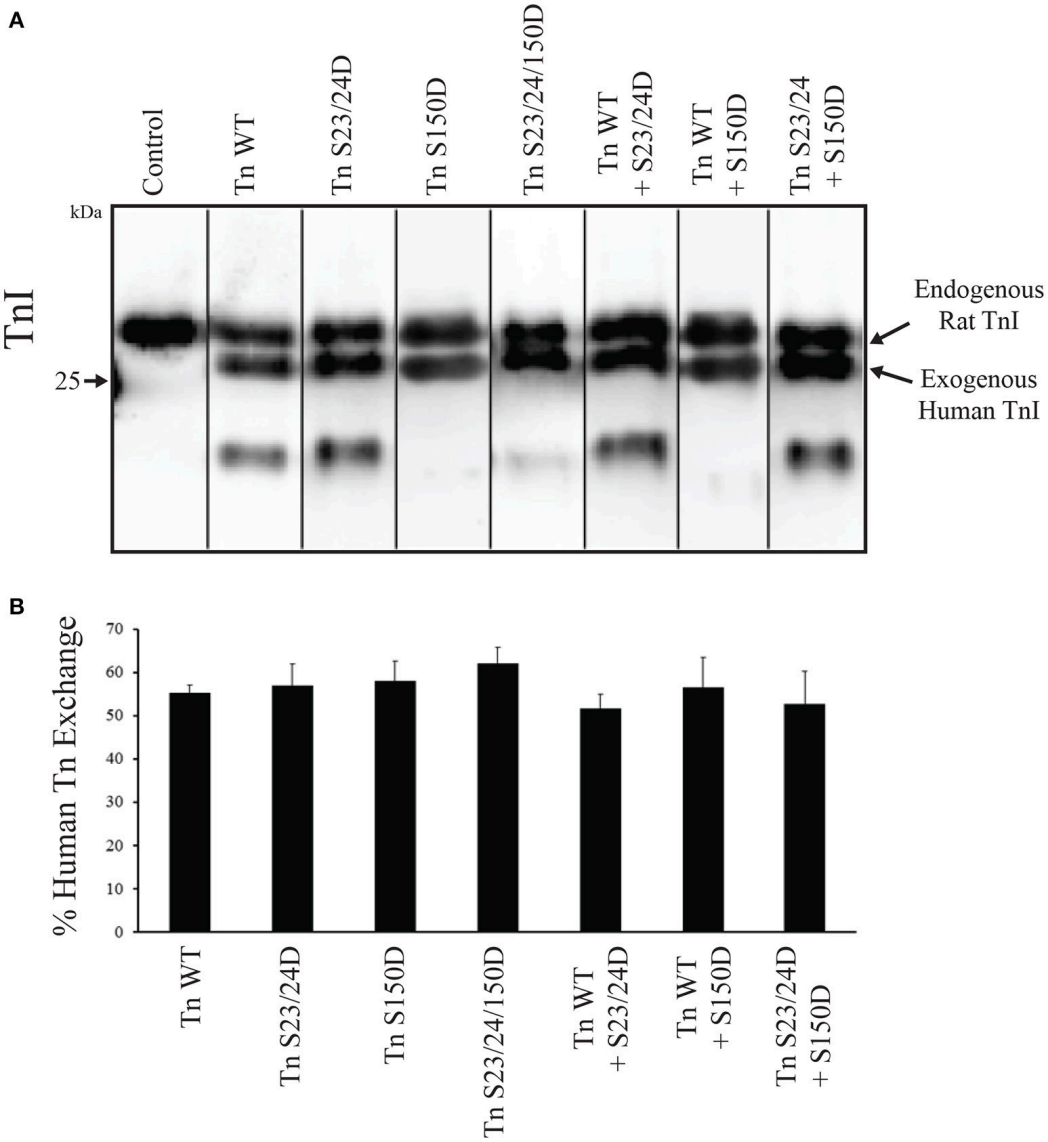
**Tn exchange into skinned cells is similar for all investigated TnI pseudo-phosphorylations. (A)** Representative TnI Western blot of myocytes following exchange demonstrates a similar presence of the faster migrating exogenous human cardiac TnI compared to the slower migrating endogenous rat cardiac TnI in all Tn's investigated. **(B)** Densitometry analysis of Western blots demonstrates the average percent of each exchanged exogenous Tn is not different. Control; purified recombinant rat WT cardiac TnI.

**Figure 4:**
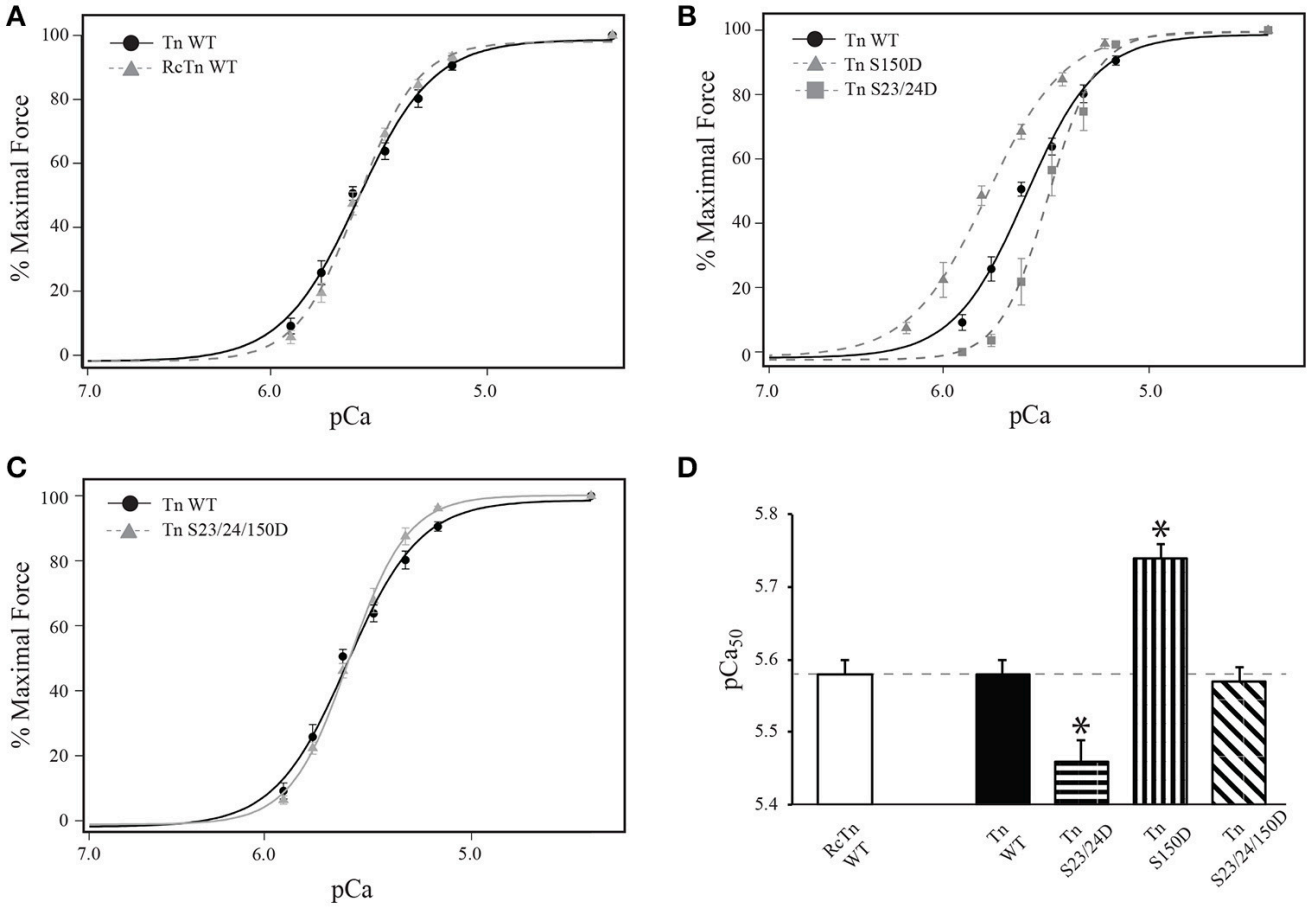
**TnI Ser-150 blunts Ser-23/24 pseudo-phosphorylation in Tn exchanged single rat cardiac myocytes**. Average normalized force-pCa measurements of Tn exchanged single cardiac myocytes. **(A)** Myocytes exchanged with WT human cardiac Tn (Tn WT: black circle, solid line; *n* = 8) or WT rat cardiac Tn (RcTn WT: gray triangle, dashed line; *n* = 8) exhibit similar Ca^2+^-sensitive force development. **(B)** The Ca^2+^-sensitive force development in myocytes exchanged with Tn containing Ser-150 pseudo-phosphorylated TnI (Tn S150D: gray triangle, dashed line; *n* = 7) is increased while Ser-23/24 pseudo-phosphorylated TnI (Tn S23/24D: gray square, dashed line; *n* = 7) is decreased compared to Tn containing WT TnI (Tn WT: black circle, solid line; *n* = 8) exchange. **(C)** The Ca^2+^-sensitive force development in myocytes exchanged with Tn containing Ser-23/24/150 pseudo-phosphorylated TnI (Tn S23/24/150D: gray triangle, solid line; *n* = 8) is identical to those with Tn WT exchange. **(D)** Comparison of pCa_50_. ^*^p < 0.05 vs. WT Tn.

Several studies have demonstrated that Ser-23/24 phosphorylation decreases Ca^2+^-sensitivity of skinned cardiac muscle preparations (Robertson et al., 1982; Zhang et al., 1995; Biesiadecki et al., 2007). Previous work from our lab demonstrated that Ser-150 phosphorylation increases Ca^2+^-sensitivity of force development in isolation and its functional integration blunts Ser-23/24 phosphorylation mediated desensitization following exchange into skinned cardiac trabeculae (Nixon et al., 2012, 2014). Previously we demonstrated the pseudo-phosphorylation of TnI at both Ser-23/24 and Ser-150 by mutation to Asp alters Ca^2+^ dependent force identical to that of actual phosphate at these sites (Biesiadecki et al., 2007; Nixon et al., 2012). To investigate the functional integration of Ser-23/24 and Ser-150 phosphorylation in the isolated skinned rat myocyte system, we measured Ca^2+^ dependent force development in left ventricular rat skinned myocytes exchanged with human cardiac Tn containing either WT TnI (Tn WT), S23/24 pseudo-phosphorylated TnI (Tn S23/24D), S150 pseudo-phosphorylated TnI (Tn S150D) or S23/24/150 pseudo-phosphorylated TnI (Tn S23/24/150D). Following exchange, the percent of exogenous Tn incorporated into all measured myocyte preparations was determined by Western blot, as the exogenous human cardiac TnI migrates faster by SDS-PAGE than endogenous rat cardiac TnI. Exchange quantification demonstrated an average of 57% exogenous Tn incorporation that was not different for any of the different Tn's exchanged (% exchange: rat Tn WT = 60.5 ± 7.5%, human WT Tn = 55.3 ± 1.8%, Tn S23/24D = 57.0 ± 5.0%, Tn S150D = 58.0 ± 4.5%, Tn S23/24/150D = 62.0 ± 3.8%; *p* > 0.05) (Figure 3). Furthermore, the exchange of human Tn WT did not alter Ca^2+^-sensitivity compared to cells exchanged with Tn containing rat WT cardiac TnI, rat cardiac Myc tagged WT TnT and rat WT TnC (RcTn WT) (Figure 4 and Table 1). Consistent with previous findings, exchange with Tn S23/24D decreased Ca^2+^-sensitivity of force development, while exchange with Tn S150D increased Ca^2+^-sensitivity compared to Tn WT (Figure 4 and Table 1) (Nixon et al., 2012). The Ca^2+^-sensitivity of cells exchanged with Tn S23/24/150D was not different from Tn WT, consistent with previous findings that S150D blunts Ser-23/24 mediated desensitization (Figure 4 and Table 1). These data demonstrate that Ser-23/24 and Ser-150 pseudo-phosphorylation impart different effects on Ca^2+^-sensitive force development in the skinned rat myocyte system, consistent with previous *in vitro* and trabeculae data (Nixon et al., 2012, 2014). Additionally, Ser-150 pseudo-phosphorylation is able to blunt the Ser-23/24 mediated desensitization when these phosphorylations occur on the same TnI molecule in the skinned rat myocyte.

**Table 1:** **Force development in skinned myocytes**.

	**Tn**	**pCa_50_**	**Hill**	**F_max_**	***n***
	RcTn WT	5.58 ± 0.01^d^^,^^e^	2.51 ± 0.13^d^	115.3 ± 13.52	8
100%	Tn WT	5.58 ± 0.02^d^^,^^e^	3.14 ± 0.14^d^	89.53 ± 10.71	8
Single Tn	Tn S23/24D	5.46 ± 0.03^a^^,^^b^^,^^c^^,^^e^	4.04 ± 0.27^a^^,^^b^^,^^c^^,^^e^	71.15 ± 6.33^b^	7
Exchange	Tn S150D	5.74 ± 0.02^a^^,^^b^^,^^c^^,^^d^	2.34 ± 0.10^d^	99.41 ± 7.68	7
	Tn S23/24/150D	5.57 ± 0.02^d^^,^^e^	3.09 ± 0.15^d^	83.00 ± 7.79	8
50/50%	Tn WT+S23/24D	5.53 ± 0.03^e^	3.41 ± 0.21^d^^,^^e^	100.67 ± 12.91	7
Dual Tn	Tn WT+S150D	5.68 ± 0.02^a^^,^^b^^,^^c^^,^^d^^,^^e^	2.15 ± 0.13^b^^,^^c^^,^^d^	82.87 ± 5.70	9
Exchange	Tn S23/24D+S150D	5.59 ± 0.02^d^^,^^e^	2.87 ± 0.30^d^	86.20 ± 4.79	7

*Values are mean ± SEM. pCa_50_, −log[Ca^2+^] at 50% force development; Hill, Hill coefficient; F_max_, maximal force in μN/mm^2^; n, number of single myocytes measured from at least three different exchanges per group. One-way ANOVA demonstrates a significant interaction. p < 0.05*.

a *Significantly different vs. Tn WT*.

b *Significantly different vs. RcTn WT*.

c *Significantly different vs. Tn S23/24/150D*.

d *Significantly different vs. Tn S23/24D*.

e *Significantly different vs. Tn S150D*.

### Ca^2+^ sensitive force development is dependent on the amount of TnI phosphorylation present

**Figure 5:**
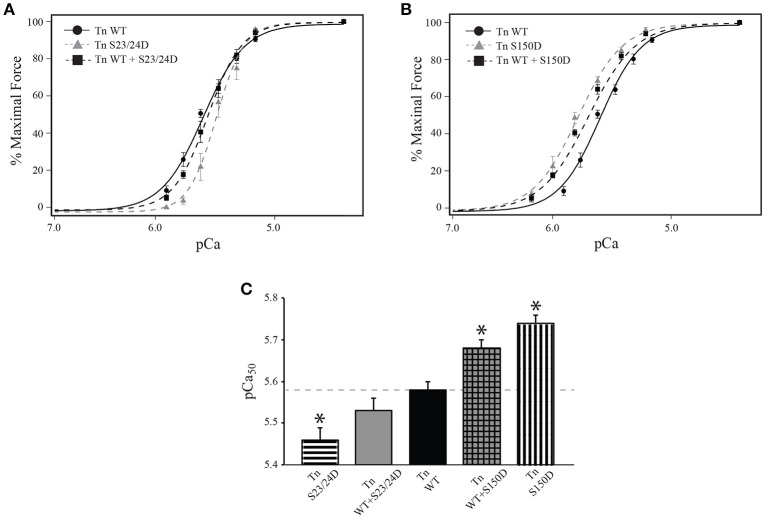
**Single myocytes exchange with 50% pseudo-phosphorylated TnI exhibit half the phosphorylation mediated sensitivity change**. Average normalized force-pCa measurements of Tn exchanged single cardiac myocytes. **(A)** The decrease in Ca^2+^-sensitive force development of myocytes exchanged with Tn containing 50% WT and 50% Ser-23/24 pseudo-phosphorylated TnI (Tn WT+S23/24D: black square, dashed line; *n* = 7) is half that of myocytes exchanged with Tn containing Ser-23/24 pseudo-phosphorylated TnI (Tn S23/24D: gray triangle, dashed line; *n* = 7) compared to WT (Tn WT: black circle, solid line; *n* = 8) exchange. **(B)** The increase in Ca^2+^-sensitive force development of myocytes exchanged with Tn containing 50% WT and 50% Ser-150 pseudo-phosphorylated TnI (Tn WT+S150D: black square, dashed line; *n* = 9) is half that of myocytes exchanged with Tn containing Ser-150 pseudo-phosphorylated TnI (Tn S150D: gray triangle, dashed line; *n* = 7) compared to Tn WT exchange. **(C)** Comparison of pCa_50_. ^*^p < 0.05 vs. WT Tn.

The functional effects of integrated TnI phosphorylations may be complexly related to the amount of TnI phosphorylation present. To determine if the relationship between Ca^2+^-sensitivity and the amount of phosphorylation present is linear for both TnI Ser-23/24 and Ser-150, we measured the Ca^2+^-dependent force development in left ventricular rat skinned myocytes. Myocytes were exchanged with a mixture of 50% human cardiac Tn WT and either 50% Tn S23/24D (Tn WT+S23/24D) or 50% Tn S150D (Tn WT+S150D). Western blot for cardiac TnI demonstrated the incorporation of 51.7 ± 3.3% WT+S23/24D and 56.5 ± 6.9% WT+S150D exogenous Tn (*p* > 0.05) (Figure 3). While we cannot directly determine the percent exchange of each exogenous Tn complex in the mixture, we assume similar exchange as we observed for Tn S23/24D and Tn S150D exchange (Figure 3). Following exchange with Tn WT+S23/24D we observed half the decrease in pCa_50_ observed for Tn S23/24D exchange compared to Tn WT exchange (change in pCa_50_ from Tn WT: Tn S23/24D = 0.11, WT+S23/24D = 0.05) (Figure 5 and Table 1). We observed similar results following exchange with Tn WT+S150D, in which the myofilament was only half as sensitized compared to exchange with Tn S150D alone (change in pCa_50_ from Tn WT: Tn S150D = 0.17, Tn WT+S150D = 0.1; *p* < 0.05) (Figure 5 and Table 1). These data demonstrate that the magnitude of phosphorylation dependent change in Ca^2+^-sensitive force development is related to the amount of TnI phosphate present at the TnI Ser-23/24 or Ser-150 residues.

### TnI Ser-23/24 and Ser-150 pseudo-phosphorylation exhibit functional integration when on different TnI molecules

**Figure 6:**
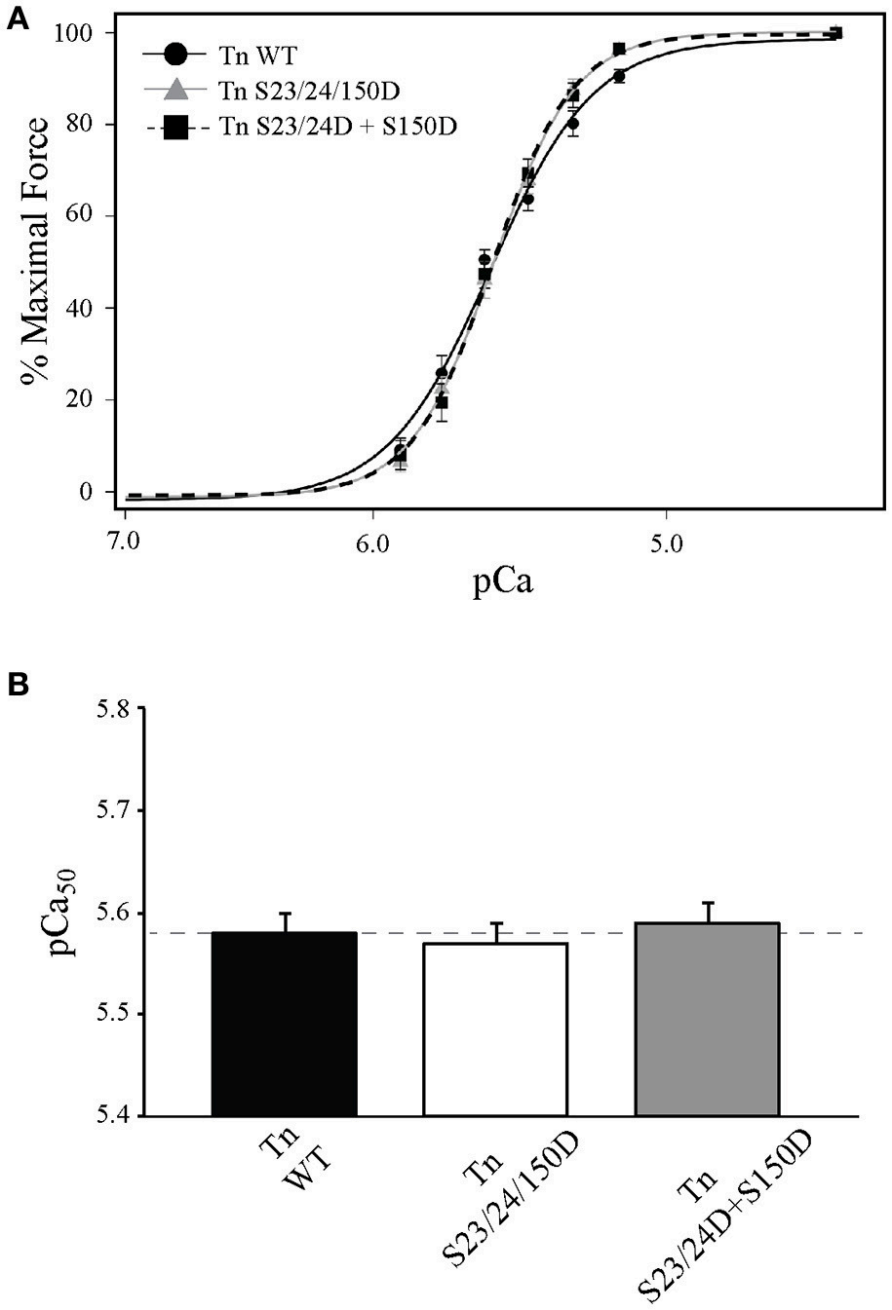
**Single myocytes exchanged with Ser-150 and Ser-23/24 pseudo-phosphorylation on different Tn exhibit Ca^2+^-sensitivity identical to both TnI pseudo-phosphorylations on the same Tn. (A)** Average normalized force-pCa measurements of Tn exchanged single cardiac myocytes exchanged with Tn containing Ser-23/24 and Ser-150 pseudo-phosphorylation on the same TnI (Tn S23/24/150D: gray triangle, solid line; *n* = 8) or exchanged with 50% Ser-23/24 and 50% Ser-150 pseudo-phosphorylated TnI on different Tn (Tn S23/24D+S150D: black square, dashed line; *n* = 7) both exhibit identical Ca^2+^ dependent force development to those exchanged with WT TnI (Tn WT: black circle, solid line; *n* = 8). **(B)** Comparison of pCa_50_.

The overwhelming majority of studies to date have primarily determined the combined function of multiple phosphorylations on the same TnI molecule. We investigated if the different molecular mechanisms utilized by Ser-23/24 and Ser-150 phosphorylation integrate when occurring on different Tn molecules. To this end, we exchanged cells with Tn containing 50% TnI S23/24D and 50% TnI S150D (Tn S23/24D+S150D) that resulted in an average of 56.8 ± 7.5 percent total exogenous Tn exchange. Thus, the Tn S23/24D+S150D exchange group is expected to result in cells containing ~25% TnI S23/24D, ~25% S150D and ~50% endogenous WT Tn in the myofilament. Calcium sensitivity of cells exchanged with Tn S23/24D+S150D was not different from those exchanged with Tn S23/24/150D, even though the pseudo-phosphorylations occurred on separate Tn molecules (Figure 6 and Table 1). Thus, these data demonstrate that pseudo-phosphorylation of TnI molecules at Ser-150 integrate to blunt the Ca^2+^ desensitizing effects of Ser-23/24 pseudo-phosphorylation even when these two phosphorylated residues are present on different TnI molecules. Therefore, the molecular mechanisms responsible for phosphorylation functional integration to differentially affect heart function can integrate across different Tn's along the myofilament.

## Discussion

The phosphorylation of different TnI residues is altered by physiological and pathological stress and therefore the combined function of multiple TnI phosphorylations plays a critical role to differentially modulate cardiac function in both the normal and diseases states. Our current study aimed to elucidate underlying mechanisms utilized by Ser-23/24 and Ser-150 phosphorylation. Our findings demonstrate that: (1) The integrated function of TnI Ser-23/24 and Ser-150 phosphorylation is dependent in part upon their regulation of different TnI protein-protein interactions within the thin filament. TnI Ser-23/24 phosphorylation alters the dissociation of the TnI C-terminus from TnC, while TnI Ser-150 phosphorylation alters binding of the TnI C-terminus to actin and slows the dissociation of Ca^2+^ from TnC in the presence of TnI. (2) TnI Ser-23/24 or Ser-150 phosphorylation occupancy linearly alters Ca^2+^-sensitive force development. (3) The mechanisms utilized by Ser-23/24 and Ser-150 phosphorylation integration to affect Ca^2+^-sensitive force development occur both when on the same and separate Tn molecules along the myofilament.

### Regulatory mechanisms of TnI Ser-23/24 and Ser-150 phosphorylation

TnI Ser-23/24 phosphorylation decreases Ca^2+^-sensitive force production (Figure 4) (Solaro et al., 1976; Kranias and Solaro, 1982; Zhang et al., 1995; Nixon et al., 2012). The cardiac TnI N-terminal region containing the Ser-23/24 residues interacts with the N-lobe of TnC (Abbott et al., 2001). The structural basis for Ser-23/24 phosphorylation to decrease Ca^2+^-sensitivity is proposed to be through alteration of this interaction between the TnI N-terminus and TnC. Peptide binding studies have supported this hypothesis by demonstrating a decreased binding affinity of TnI N-terminal peptides that contain phosphorylated Ser-23/24 to TnC (Ferrières et al., 2000). NMR studies also complement these findings, demonstrating the TnI N-terminus makes contacts with the N-terminal domain of TnC near the regulatory Ca^2+^-binding site (Finley et al., 1999; Gaponenko et al., 1999; Abbott et al., 2000). These contacts are altered when Ser-23/24 are substituted with phosphomimetics (Finley et al., 1999; Ward et al., 2004; Hwang et al., 2014). The mechanisms by which alteration of TnI N-terminus binding to TnC is propagated through the Tn complex to affect Ca^2+^-sensitivity remain unclear. Current data supports that the Ser-23/24 phosphorylation can act either by directly destabilizing Ca^2+^ binding to the TnC regulatory site or indirectly by modulating binding affinities of the TnI C-terminal region to TnC or actin (switch peptide or inhibitory peptide binding, respectively). Data from our model and binding studies suggest that Ser-23/24 phosphorylation acts, at least in part, by altering the TnI-TnC interaction to increase the dissociation of TnI from TnC-Ca^2+^ (Figure 1). Altering the dissociation of TnI from TnC-Ca^2+^ is sufficient to recapitulate Ser-23/24 phosphorylation biochemical data and is consistent with a proposed intra-molecular interaction between TnI subdomains, whereby Ser-23/24 phosphorylation places the N-terminus in a conformation to interact with the basic residues on the C-terminal TnI regulatory region near the switch peptide (Howarth et al., 2007; Warren et al., 2009).

TnI Ser-150 phosphorylation increases Ca^2+^-sensitive force production (Buscemi et al., 2002; Nixon et al., 2012; Oliveira et al., 2012). This Ser-150-mediated increase in myofilament Ca^2+^-sensitivity has been proposed to occur by modulating the affinity of the TnI inhibitory peptide binding to actin and/or switch peptide binding to the TnC N-lobe (Ouyang et al., 2010; Nixon et al., 2012). The location of Ser-150 immediately adjacent to the TnI inhibitory peptide has potential for phosphate incorporation to repel the TnI C-terminus from actin and/or promote the binding of the switch peptide to TnC. Enhanced TnC-TnI switch peptide binding is supported by FRET experiments indicating that Ser-150 pseudo-phosphorylation shortens inter-site distances between TnI and TnC (Ouyang et al., 2010). In addition, data from our lab demonstrating that Ser-150 phosphomimetic substitution increases Ca^2+^-sensitivity in isolated Tn (Nixon et al., 2014), supporting that Ser-150 phosphorylation enhances the binding of the TnI switch peptide to TnC in the absence of interactions with the actin filament. Enhanced binding of the switch peptide to TnC would contribute to a stabilization of the TnC-Ca^2+^ affinity and a decrease in Ca^2+^ dissociation, as suggest by our model (Figure 1). Current findings from our model further suggest that Ser-150 phosphorylation destabilizes the interaction of the TnI C-terminus with actin to promote thin filament activation. This mechanism is validated by our solid-phase protein binding experiments in which Ser-150 pseudo-phosphorylation decreases the binding affinity of isolated TnI to actin (Figure 2). Thus, we propose that the prominent TnI affinities acted upon by Ser-23/24 and Ser-150 phosphorylation differ in that phosphorylated Ser-23/24 alters the affinity of the switch peptide for TnC by accelerating TnI dissociation from TnC-Ca^2+^. In contrast, while Ser-150 phosphorylation may alter other TnI affinities in the thin filament, we demonstrate that the decreased affinity of pseudo-phosphorylated TnI at Ser-150 for actin is sufficient to elicit the observed increased calcium sensitivity.

Although the structural basis for the integrated effects of TnI of Ser-150 and Ser-23/24 phosphorylation remains unclear, our data provides significant mechanistic insight into this functional integration. Previous work indicates the TnI C-terminus is located in close proximity to the TnI N-terminus in the activated Tn complex (Howarth et al., 2007; Warren et al., 2009). This proximity of the TnI N-terminus containing Ser-23/24 to the C-terminus containing Ser-150 provides a mechanism by which Ser-150 and Ser-23/24 phosphorylation may interact to produce an integrated effect on Ca^2+^-sensitivity when on the same TnI molecule (Nixon et al., 2012). Our current data, however, demonstrates that phosphorylation of Ser-150 and Ser-23/24 on the same TnI molecule is not necessary to produce the Ca^2+^-sensitivity blunting effect (Figure 5). Although a direct TnI N-C terminal interaction may occur, we demonstrate Ser-23/24 and Ser-150 phosphorylation functional integration also utilizes separate, distinct molecular mechanisms that modulate TnI protein-protein interactions within the thin filament to regulate heart function. These data present a model in which a cumulative effect of each Tn phosphorylation along the thin filament must be considered to contribute to the overall observed Ca^2+^-sensitivity and contractile effects.

### TnI phosphorylation functional integration effects on myofilament regulation

A large subset of the literature has described how isolated Tn modifications affect Ca^2+^-mediated force development. The phosphorylation of TnI is significant considering that altered Ca^2+^ sensitivity has been shown to underlie physiological modulation of cardiac output as well as potential adaptive and maladaptive responses in cardiac dysfunction (Zakhary et al., 1999; Kobayashi and Solaro, 2005; Zhang et al., 2012; Lang et al., 2015). While Ser-150 has been shown to be phosphorylated by p-21 activated kinase and AMP-activated protein kinase (AMPK) *in vitro*, AMPK seems to be the likely relevant kinase *in vivo* (Buscemi et al., 2002; Sheehan et al., 2009; Oliveira et al., 2012). Previous studies have demonstrated AMPK associates with TnI and phosphorylates Ser-150, and isolated hearts perfused with an AMPK activator increase Ser-150 phosphorylation (Nixon et al., 2012; Oliveira et al., 2012). Several reports have demonstrated the importance of AMPK signaling in cardiac metabolism and contractility. Knockout or inhibition of AMPK has been shown to lead to cardiomyopathy and/or altered contraction concurrent with decreases in TnI Ser-150 phosphorylation (Chen et al., 2014; Sung et al., 2015). It remains to be resolved if the pathological effects of AMPK inactivation result from metabolic dysregulation, contractile deficiencies or both. We have demonstrated the effect of Ser-150 phosphorylation to increase myofilament calcium sensitivity of force development in skinned trabeculae, increase steady-state calcium binding to isolated troponin, and slow calcium dissociation from TnC on the thin filament (Nixon et al., 2012, 2014). Additionally, significant evidence has pointed to a role for Ser-150 phosphorylation under pathophysiologic conditions. Ser-150 phosphorylation is increased following acute ischemia in the murine heart and the calcium sensitization induced by Ser-150 phosphorylation was shown to blunt myofilament calcium desensitization caused by acidic pH (Nixon et al., 2014). As such, Ser-150 phosphorylation may play an important role to increase calcium sensitivity and force development resulting in preserving myocardial function under pathological stress during ischemia. The functional integration between Ser-23/24 and Ser-150 phosphorylation also presents a potentially important interplay *in vivo*. Previous work has demonstrated chronic beta adrenergic stimulation results in increased TnI Ser-150 phosphorylation (Taglieri et al., 2011). Similarly, both Ser-23/24 and Ser-150 phosphorylation are increased during myocardial ischemia (Nixon et al., 2014). Together these findings suggest cross-talk between beta adrenergic and AMPK mediated signaling to increase cardiac output by accelerating thin filament deactivation/muscle relaxation while maintaining normal force development. Future work is needed to investigate the signaling cross-talk between B-adrenergic and AMPK signaling, functional integration of Ser-23/24 and Ser-150 phosphorylation and their effects on *in vivo* cardiac contractility and energy metabolism.

Whether these isolated functional effects of Ser-150 phosphorylation are maintained in the integrated network alongside other phosphorylation events remains understudied. Our previous work demonstrated functional integration of TnI Ser-150 with Ser-23/24 phosphorylation blunted the myofilament desensitization induced by TnI Ser-23/24 phosphorylation alone (Figure 4) (Nixon et al., 2012, 2014). In the current study we assessed the contribution of TnI Ser-23/24 and Ser-150 integrated TnI phosphorylation by analyzing their effects on Ca^2+^-sensitive force development. We demonstrate that the overall observed Ca^2+^-sensitivity is proportional to the amount of phosphorylated TnI present at up to ~ 57% exogenous Tn exchange (Figure 3). Although we did not achieve 100% exchange of exogenous Tn into our single myocyte system, previous studies have suggested 50–60% exchange is sufficient to elicit maximal effects on Ca^2+^-sensitivity for Ser-23/24 phosphorylation (Wijnker et al., 2013). Thus, it is possible that the proportional functional effects of TnI phosphorylation exist only within a linear range of exchange below 60%. In addition, while we detected a degradation fragment of TnI in our exchanged cells, the presence of this fragment was variable amongst TnI exchange groups and did not appear to correlate with changes in pCa50 nor maximal tension values. The appearance of a TnI cleaved product is consistent with previous studies demonstrating that approximately 13% of the total TnI exists as this TnI fragment in the normal rat heart (Yu et al., 2001; Barbato et al., 2005). Nevertheless, because these changes in Ca^2+^-sensitive force development are linear with the amount of phosphorylation present, our data suggests that this is not a mechanism contributing the different functional effects of Ser-23/24 and Ser-150 phosphorylation upon integration. As such, quantification of multiple TnI phosphorylations is necessary to infer the effect of TnI phosphorylation on the overall observed Ca^2+^-sensitivity in its relation to force production.

Previously we demonstrated functional integration of TnI Ser-23/24 and Ser-150 pseudo-phosphorylation occurred when both phosphorylations were on the same TnI molecule (Nixon et al., 2012, 2014). We now demonstrate that TnI Ser-23/24 and Ser-150 pseudo-phosphorylation integration similarly affects steady-state Ca^2+^-sensitive force development whether these phosphorylations are on the same or different TnI molecules (Figure 6). This suggests that the TnI Ser-23/24 and Ser-150 phosphorylation integration of Ca^2+^-sensitive force development function occurs through a cumulative effect of separate Tn contributions along the thin filament. Our current data further demonstrates that these phosphorylations exhibit complex functional integration both when on the same TnI molecule as well as when on different TnI molecules. These relationships may not be universal for other measurements of myofilament function, such as maximal force, Ca^2+^ dissociation, length dependent activation, etc. and may be further dependent upon the specific TnI phosphorylation. In fact, we previously demonstrated TnI Ser-23/24 and Ser-150 phosphorylation integration functions to differently alter Ca^2+^-sensitivity and Ca^2+^ dissociation. That is, while the integration of Ser-23/24 and Ser-150 exhibit expected Ca^2+^-sensitivity, Ca^2+^ dissociation remains unexpectedly accelerated (Nixon et al., 2012, 2014). Likewise, we have also demonstrated the functional integration of TnI Ser-23/24 with Tyr-26 phosphorylation, where the combination of these phosphorylations does not exhibit an expected effect on Ca^2+^-sensitivity but does exhibit the expected further accelerated Ca^2+^ dissociation (Salhi et al., 2014). Further experiments are ultimately needed to define the integrated effects of other TnI phosphorylations on these different functional measurements.

In all, our current study demonstrates that the apparent magnitude and rates of myofilament activation and deactivation are dependent on multiple mechanisms functioning within Tn that comprise intricate protein-protein interactions. We demonstrate different TnI phosphorylations can affect varied mechanisms within Tn that differentially alter specific contractile functions (Ca^2+^ binding, kinetics, force development, etc.). It is therefore the combination of each TnI phosphorylation's affect on different mechanisms within Tn to alter myofilament activation that imparts functional integration. The integrative role of multiple TnI phosphorylations thus allows for the complex fine-tuning of cardiac function through Tn.

## Conclusion

Ultimately, TnI regulation of cardiac function is dependent upon the homeostatic balance between the mechanistic protein-protein interactions of each phosphorylation on Tn units along the myofilament. Our findings demonstrate that the different effects of Ser-23/24 and Ser-150 phosphorylation upon functional integration combination are the result of distinct Tn mediated mechanisms that alter different Ca^2+^ regulated TnI interactions within the thin filament. We further demonstrate that the blunting effect of TnI Ser-150 phosphorylation integration on Ser-23/24 dependent desensitization is recapitulated in the single myocyte system of Ca^2+^-sensitive force development and this effect of phosphorylation on Ca^2+^-sensitivity is proportional to the amount of TnI phosphorylation present. Finally, our data demonstrates that TnI phosphorylations with divergent properties can functionally interact while on separate Tn molecules along the myofilament to produce a complex modulation of overall observed Ca^2+^-sensitive force production. To fully understand the contractile outcome of multiple TnI phosphorylations it is therefore important to investigate TnI phosphorylation alterations in their integrated state as they occur in the heart.

## Author contributions

HS: Conducted experiments, analyzed data and wrote the manuscript; NH: Conducted experiments and analyzed data; JS: Conducted experiments and edited the manuscript; EB: Conducted experiments and edited the manuscript; MZ: Contributed to design and edited the manuscript; PJ: Contributed to design and edited the manuscript; JD: Contributed to design and edited the manuscript; BB: Contributed to design, oversaw experiments, wrote and edited the manuscript.

## Funding

Support for this work was obtained from NIH grants HL114940 (to BB), HL114940-S1 (to HS), HL091986 (to JD), HL113084 (to PJ) and American Heart Association grant GRNT27760114 (To MZ).

### Conflict of interest statement

The authors declare that the research was conducted in the absence of any commercial or financial relationships that could be construed as a potential conflict of interest.

## Abbreviations

Calcium: Ca^2+^
Tn: troponin
TnI: troponin I
WT: wild-type.
